# Recent Perceived Stress, Amygdala Reactivity to Acute Psychosocial Stress, and Alcohol and Cannabis Use in Adolescents and Young Adults With Bipolar Disorder

**DOI:** 10.3389/fpsyt.2021.767309

**Published:** 2021-11-15

**Authors:** Vanessa Le, Dylan E. Kirsch, Valeria Tretyak, Wade Weber, Stephen M. Strakowski, Elizabeth T. C. Lippard

**Affiliations:** ^1^Department of Psychiatry and Behavioral Sciences, Dell Medical School, University of Texas, Austin, TX, United States; ^2^Waggoner Center for Alcohol and Addiction Research, University of Texas, Austin, TX, United States; ^3^Institute for Neuroscience, University of Texas, Austin, TX, United States; ^4^Department of Psychology, University of Texas, Austin, TX, United States; ^5^Institute of Early Life Adversity Research, University of Texas, Austin, TX, United States

**Keywords:** bipolar disorder, alcohol drinking, cannabis, stress-psychological, magnetic resonance imaging (MRI)

## Abstract

**Background:** Psychosocial stress negatively affects the clinical course of bipolar disorder. Studies primarily focused on adults with bipolar disorder suggest the impact of stress is progressive, i.e., stress response sensitizes with age. Neural mechanisms underlying stress sensitization are unknown. As stress-related mechanisms contribute to alcohol/substance use disorders, variation in stress response in youth with bipolar disorder may contribute to development of co-occurring alcohol/substance use disorders. This study investigated relations between psychosocial stress, amygdala reactivity, and alcohol and cannabis use in youth with bipolar disorder, compared to typically developing youth.

**Methods:** Forty-two adolescents/young adults [19 with bipolar disorder, 23 typically developing, 71% female, age_mean_ ± SD = 21 ± 2 years] completed the Perceived Stress Scale (PSS), Daily Drinking Questionnaire modified for heaviest drinking week, and a modified Montreal Imaging Stress functional MRI Task. Amygdala activation was measured for both the control and stress conditions. Main effects of group, condition, total PSS, and their interactions on amygdala activation were modeled. Relationships between amygdala response to acute stress with recent alcohol/cannabis use were investigated.

**Results:** Greater perceived stress related to increased right amygdala activation in response to the stress, compared to control, condition in bipolar disorder, but not in typically developing youth (group × condition × PSS interaction, *p* = 0.02). Greater amygdala reactivity to acute stress correlated with greater quantity and frequency of alcohol use and frequency of cannabis use in bipolar disorder.

**Conclusion:** Recent perceived stress is associated with changes in amygdala activation during acute stress with amygdala reactivity related to alcohol/cannabis use in youth with bipolar disorder.

## Introduction

Individuals with bipolar disorder show a higher prevalence of alcohol/substance use disorders than the general population. Up to 60% of individuals with bipolar disorder will present with comorbid alcohol/substance use disorders at some point in their lifetime (1, 2). An understanding of the neurophysiological biomarkers of risk could inform novel prevention and early intervention strategies. Despite the known devastating consequences of this comorbidity (3), our knowledge of the biological mechanisms that contribute to increased risk for comorbid alcohol/substance use disorders in bipolar disorder is limited. Several groups reported that structural and functional differences in anterior-paralimbic brain networks are related to risk for alcohol initiation and development of alcohol/substance misuse and problems over time (4–9). These studies excluded youth with bipolar disorder. We recently completed a longitudinal study identifying that the structure of anterior-paralimbic regions were associated with initiation and risk for future alcohol/cannabis use problems in bipolar disorder. Specifically, we found more significant abnormalities in structure of anterior-paralimbic regions in youth with bipolar disorder that prospectively develop alcohol/cannabis use problems, compared to youth with bipolar disorder who do not (10). How these structural differences functionally translate into risk for alcohol/substance use disorders is unknown, but research suggests it may relate to their role(s) in stress response.

The anterior-paralimbic system implicated in bipolar disorder (11) is also involved in the stress response. Changes in amygdala, insula, rostral and ventral prefrontal cortex (PFC) activation have been observed following stress and associated with cortisol response (12–15). These studies, however, focused on typically developing adults and excluded individuals with bipolar disorder. There is limited data investigating neural responses to stress in bipolar illness (16, 17). While these studies suggest amygdala differences may underlie stress sensitivity in bipolar disorder, they did not investigate how variation in neural response relates to clinical features, such as alcohol/cannabis use. Associations between brain stress systems and alcohol/substance use disorders are predominantly supported by studies in individuals with alcohol/substance use disorders (18, 19). Alcohol/substance misuse often emerges in young adulthood, and differences in how young adults respond to stress may represent one biomarker capable of predicting risk of alcohol/substance use disorders (15, 20), yet prior studies were limited to adults (16, 17). Studies in adults with bipolar disorder suggest stress sensitivity is progressive—i.e., stress responses sensitize with age—and may differ depending on clinical subtypes (21). Specifically, sensitization has been proposed to underlie the progression of bipolar disorder and alcohol/substance use disorders (22–26). It is unknown if differences in neural responses to stress are potentiated by recent stress in adolescence/young adulthood in bipolar disorder, nor if stress potentiation impacts the clinical course of bipolar disorder, i.e., may contribute to greater alcohol/substance use, early in illness course.

### The Current Study

This report is a secondary analysis of a previously published dataset (27) in which results suggested differences in stress response early in illness course in bipolar disorder, compared to typically developing adolescents/young adults, and suggested variation in neural response to stress relates to substance use and mood symptom recurrence. While the primary analysis did not observe between-group differences in amygdala activation, this secondary analysis sought to investigate if recent perceived stress relates to variation in amygdala response to acute stress. Based on proposed stress sensitization models in bipolar disorder, we hypothesized recent perceived stress would be associated with greater increases in amygdala response to acute stress in bipolar disorder, compared to typically developing adolescents/young adults, with these interactions contributing to hyperactive stress response over time. To begin testing this hypothesis, we modeled interactions between group, recent perceived stress, and stress response [during the Stress Math Task (SMT)] on amygdala activation. As stress sensitization is thought to contribute to alcohol and drug use behavior and development and maintenance of alcohol/substance use disorders (22–24), we investigated if variation in amygdala response to acute stress was associated with greater recent alcohol/cannabis use.

## Materials and Methods

### Participants

Participants included 23 typically developing adolescents/young adults and 19 adolescents/young adults with bipolar disorder type I. The Structured Clinical Interview for Diagnostic and Statistical Manual of Mental Disorders (DSM-5) Research Version (SCID-5-RV) (28) was used to confirm psychiatric diagnoses. Current mood symptoms were assessed using the Hamilton Depression Rating Scale (HDRS) (29) and Young Mania Rating Scale (YMRS) (30). No individuals had greater than mild mood symptoms at the time of their scan (i.e., HDRS scores were ≤ 15; YMRS scores were ≤ 18). The Wechsler Abbreviated Scale of Intelligence-Second Edition (WASI-II) was used as a measure of full-scale intelligence quotient (FSIQ-2), and participants with IQ <85 were excluded. Other exclusion criteria included a positive pregnancy test, history of severe alcohol/substance use disorder, history of major medical illness with possible neurological or central nervous system outcomes, or a medical condition or previous surgery preventing participation in magnetic resonance imaging (MRI) scanning. We did not exclude participants with mild or moderate alcohol/substance use disorders to avoid recruitment of super healthy groups and to increase generalizability of findings (31) as we aimed to investigate stress response related to alcohol misuse. Typically developing participants were also excluded if they had a history of mood, psychosis, or anxiety disorders, lifetime suicide attempt, or history of psychotropic medication use. Urinalysis was conducted to assess for substance use and pregnancy on the day of MRI assessment. All participants were asked to not consume alcohol or drugs for the day preceding the MRI scan. A full description of this dataset has been published in a prior manuscript (27). The University of Texas at Austin Institutional Review Board approved all study procedures and all participants provided written consent prior to study participation. All data was collected prior to March 2020 (before the COVID-19 pandemic associated mandates and guidelines emerged in the United States).

### Measures

#### Recent Perceived Stress

Recent perceived stress was measured using the Perceived Stress Scale (PSS) (32). This 10-item questionnaire assesses the degree to which individuals considered different experiences over the past month stressful. Each item asked how often different feelings and thoughts (e.g., been upset because something happened unexpectedly) occurred in the past month using a 5-point Likert scale, with 0 being “never” and 4 as “very often.” Higher total scores equate to greater perceived stress over the past month.

#### Recent Alcohol/Substance Use

The Daily Drinking Questionnaire (DDQ) (33) modified for heaviest drinking week (DDQ-H) was used to assess recent alcohol use. Participants were asked to report the number of drinks consumed each day for their heaviest drinking week over the past month. Total drinking days and total drinks per heaviest drinking week were calculated. The Daily Drug-Taking Questionnaire (DDTQ) was used to assess percentage of participants per group that used cannabis or tobacco products over the past month as well as total number of cannabis use days during heaviest drug-taking week in those reporting cannabis use [(34); Parks, 2001 (unpublished manuscript)].

### MRI Acquisition

A high-resolution sagittal structural MRI scan was acquired using a 32-channel head coil with a three-dimensional gradient echo T1-weighted sequence on a 3-Tesla Siemens Skyra (Seimens, Erlangen, Germany) with the following parameters: repetition time (TR) = 1,900 ms, echo time (TE) = 2.42 ms, matrix = 224 × 224, field of view = 220 × 220 mm^2^, 192 one-mm slices without gap and one average. A single-shot echo-planar imaging sequence aligned with the anterior-posterior commissure plane was used for fMRI data with the following parameters: multiband factor = 3, TR = 2,000 ms, TE = 30 ms, matrix = 128 × 128, field of view = 220 × 220 mm^2^, and 72 two-mm slices without gap.

### Stress Math Task (SMT)

Participants completed the SMT, a modified version of the Montreal Imaging Stress Task (MIST) (35, 36), which includes a control condition of 40 math problems and two answer choices and a stress condition of 80 more difficult math problems and three answer choices (37, 38). Each math problem was presented for 5 s with a 1.5 s inter-trial interval, during which a fixation point was presented, between problems. Participants had 5 s to answer math problems using a button box. During the stress condition, while problems were presented for 5 s, mirroring the control condition, participants were told they had between 1 and 3 s to choose their answer so they had to respond more quickly while still maintaining accuracy. Additionally, the stress condition included six pre-recorded negative auditory feedback messages regarding their performance that were presented at fixed time points during inter-trial intervals. Feedback was presented to all participants regardless of performance. A MRI safe pulse oximeter was used to record participants' heart rates throughout the scan to assess physiological response to the stress condition. All participants completed the control condition first, followed by the stress condition. Following task completion, participants were debriefed and informed that their performance was not evaluated.

### Functional MRI Data Preprocessing

FMRI data was preprocessed with Statistical Parametric Mapping software (SPM12; http://www.fil.ion.ucl.ac.uk/spm). Briefly, data was realigned, corrected for slice timing, coregistered to anatomical data, spatially normalized to the T1-weighted template image, and spatially smoothed with a 4 mm FWHM Gaussian kernel. The WFU PickAtlas Tool (http://www.fmri.wfubmc.edu/download.htm) in SPM12 was used to define bilateral amygdala *a priori* regions of interest (ROIs). Event-related response amplitudes were estimated at the subject level for control and stress condition math problems using the general linear model. Onset and duration of each math problem during control and stress conditions (separately) were defined as events and compared to the inter-trial intervals during the control and stress conditions, respectively. Events during the stress condition were only compared to inter-trial intervals when no audio recording was presented. Estimated six parameter spatial transformation from realignment was included as a task regressor. Bilateral amygdala event amplitude was calculated and extracted for both the control and stress conditions and used for statistical analysis.

### Statistical Analysis

#### Between-Group Differences in Demographics and Clinical Factors, Heart Rate, Task Performance, and Recent Perceived Stress

Between-group differences in the demographic and clinical variables were previously published and are summarized in Table 1 for convenience. Briefly, continuous data were assessed with a *t*-test or Wilcoxon, as appropriate. Categorical variables were assessed with Chi square or Fisher's exact, as appropriate. Similarly, between group differences and main effect of stress condition and group by stress condition interactions on heart rate and task performance during the SMT were previously published. Results are summarized below. Between group difference in PSS total score was modeled, covarying age, and sex.

**Table 1:** Demographic and clinical factors stratified by group.

		**Typically developing**	**Bipolar disorder**	***p*-value**
		**(*N* = 23)**	**(*N* = 19)**	
Demographics	Mean age (SD)	21.1 (1.9)	21.4 (2.2)	0.62
	Number of females (%)	16 (70)	14 (74)	1.00^f^
	Mean WASI-II FSIQ-2^a^	119 (12)	116 (9)	0.36
Mood scales, perceived stress,	HDRS (SD)^b^	2 (3)	9 (4)	<0.0001^z^
and illness duration	YMRS (SD)^c^	1 (1)	1 (3)	0.72^z^
	PSS (SD)^d^	22 (7)	32 (8)	<0.0001
	Illness duration (SD)^e^	N/A	3.7 (2.1)	N/A
Alcohol/cannabis use disorders	Current cannabis use disorder, mild (%)	2 (9)	3 (16)	0.64^f^
	Current cannabis use disorder, moderate (%)	1 (4)	0 (0)	1.00^f^
	Past cannabis use disorder, mild (%)	0 (0)	1 (5)	0.45^f^
	Past alcohol use disorder, mild (%)	2 (9)	4 (21)	0.38^f^
Past month alcohol/cannabis use	Total drinks (SD)^f^	10.9 (7.6)	8.7 (7.8)	0.38
	Number of drinking days (SD)^f^	2.6 (2)	2.8 (2)	0.71
	Cannabis users (%)^g^	7 (30%)	10 (53%)	0.14
	Number of cannabis use days (%)^g^^,^ ^h^	3.7 (2.9)	4.1 (2.9)	0.80^z^
	Tobacco users (%)^g^	1 (0.04)	6 (32)	0.03^f^
Urinalysis toxicology screen	Tetrahydrocannabinol (%)	5 (22)	6 (32)	0.50^f^
	Amphetamines (%)	1 (4)	2 (11)	0.58^f^
	Benzodiazepines (%)	1 (4)	1 (5)	1.00^f^
	Phencyclidines (%)	0 (0)	1 (5)	0.45^f^

*Between-group (bipolar disorder vs. typically developing) differences in age, FSIQ-2, Total Drinks/Heaviest Drinking Week, Number of Drinking Days/Heaviest Drinking Week were compared using a two-sample t-test. All other factors were examined with a Mann–Whitney–Wilcoxon or Fisher Exact tests, as appropriate*.

*^f^represents p-value calculated with Fisher exact test*.

z *represents p-value calculated with a Mann–Whitney–Wilcoxon Test*.

a *FSIQ-2 represents the composite score for the full-scale intelligence quotient comprising verbal comprehension and matrix reasoning subtests on the Wechsler Abbreviated Scale of Intelligence-Second Edition (WASI-II)*.

b *Past week depression symptoms were measured using the Hamilton Depression Rating Scale (HDRS)*.

c *Past week mania symptoms were measured using the Young Mania Rating Scale (YMRS)*.

d *Past month perceived stress was measured using the Perceived Stress Scale (PSS)*.

e *Illness Duration was determined by calculating the time (years) between first manic episode and age at fMRI scan*.

f *Recent alcohol use was measured with the Daily Drinking Questionnaire adapted for the heaviest week over the past 30 days (DDQ-H)*.

g *Recent cannabis and tobacco use was measured with the Daily Drug-Taking Questionnaire adapted for the heaviest week over the past 30 days (DDTQ-H)*.

h *Mean number of cannabis use days in individuals reporting past month cannabis use*.

#### Amygdala Reactivity During SMT and Recent Perceived Stress

To investigate activation changes in response to stress, main effects of group (bipolar and typically developing), condition (control and stress), total PSS, and their interactions were modeled, with amygdala activation during the control and stress conditions of the SMT as a repeated within subject variable. Biological sex and age were included as covariates in all models. Following a significant group by condition by total PSS interaction, models were repeated, stratified by group. Following a significant total PSS by condition interaction, change in amygdala activation to stress (stress condition minus control condition) was calculated for each individual and within group models were repeated with change in amygdala as the dependent variable to facilitate interpreting PSS by condition interactions. Any significant models were repeated for sensitivity analyses after covarying (separately) task accuracy (i.e., number of incorrect responses during the stress condition), tobacco use (yes/no), positive toxicology screen (yes/no), and total HDRS scores. Additionally, we explored relations between time since first manic episode and neural responses to stress (calculated amygdala activity during stress minus control condition), covarying sex and age, in bipolar disorder. Significance was defined as alpha <0.05 for these planned analyses.

#### Amygdala Reactivity During SMT and Relations With Alcohol/Cannabis Use

Change (stress condition minus control condition) in amygdala ROI activation relations with frequency and quantity of recent alcohol use were investigated. We only assessed a respective hemisphere of the ROI if it showed a significant group × PSS × condition interaction above. Specifically, group, change in amygdala activation to the stress condition, and interactions between these variables were modeled with total drinking days (frequency) and total drinks consumed (quantity) during heaviest drinking week over the past month as the dependent variables (modeled separately). Following a significant group by change in amygdala activation interaction on frequency or quantity of recent alcohol use, models were repeated, stratified by group. Biological sex and age were included as covariates in all models. Significance was defined as alpha <0.05. Parallel models were repeated with number of days using cannabis during heaviest drug-taking week as the dependent variable.

## Results

### Between-Group Differences in Demographics and Clinical Factors, Heart Rate, Task Performance, and Recent Perceived Stress

As previously published (27), the bipolar disorder group showed greater HDRS scores and had more individuals who used tobacco over the past month compared to the typically developing group. No other between group differences in demographic or clinical characteristics were observed. Between-group differences in the demographic and clinical variables are summarized in Table 1. Across all participants, the stress condition of the SMT, compared to the control condition, was associated with an increase in heart rate (main effect of condition: *p* = 0.002), increase in response time (main effect of condition: *p* = 0.0001), and decrease in task accuracy (main effect of condition: *p* = 0.0001). A main effect of group on accuracy was observed (*p* = 0.04), with bipolar participants making more errors compared to typically developing participants. Total PSS scores were normally distributed in both groups (typically developing group minimum-max PSS scores: 11–40; bipolar group: 15–44). The bipolar disorder group demonstrated significantly higher PSS scores, compared to the typically developing group (see Table 1).

### Amygdala Reactivity During SMT and Recent Perceived Stress

**Figure 1:**
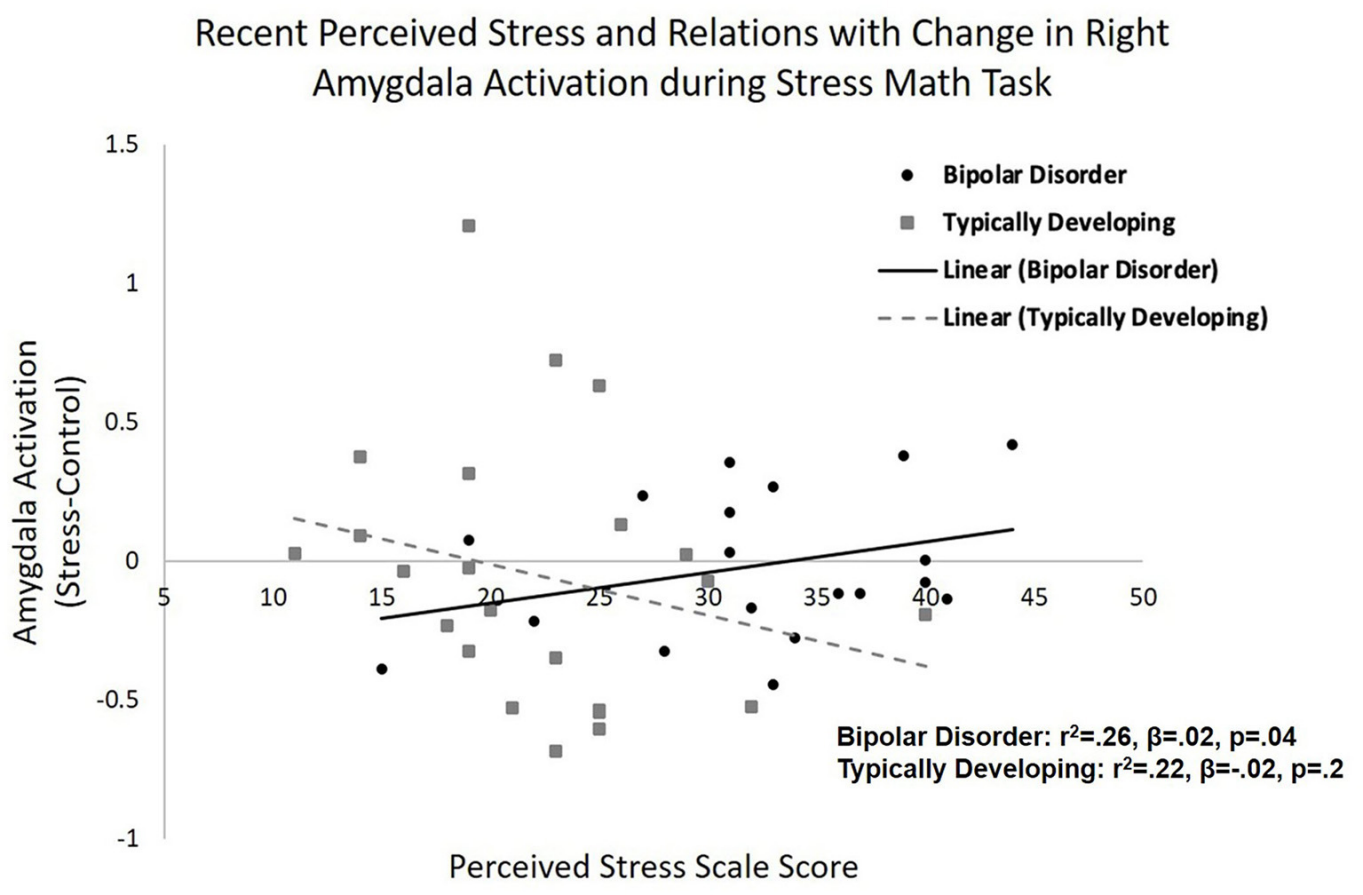
Amygdala reactivity to acute psychosocial stress and associations with recent perceived stress. Relations between recent perceived stress (PSS) and right amygdala reactivity to acute psychosocial stress [stress condition minus control condition during stress math task (SMT)] in typically developing young adults and young adults with bipolar disorder. A group by condition by PSS interaction was observed (*p* = 0.02). Greater PSS related to increased right amygdala activation in response to the stress condition, compared to control condition, in bipolar disorder (*p* = 0.04), but not in typically developing adolescents/young adults (*p* = 0.2).

Using a mixed model analysis, with condition as a repeated within subject factor, there was a group by condition by total PSS interaction (β = 0.15, p = 0.024). When stratifying by group, young adults with bipolar disorder exhibited a condition by total PSS interaction (β = 0.33, *p* = 0.04; Figure 1). Specifically, greater perceived stress was associated with increased right amygdala activation in response to the stress condition (*r*^2^ = 0.26, *p* = 0.04) in bipolar disorder. The typically developing group did not show a condition by total PSS interaction (β = 0.1, *p* = 0.2). There were no significant effects when investigating left amygdala activation. The group by condition by total PSS interaction on right amygdala activity remained significant when controlling for task accuracy, i.e., number of incorrect problems (*p* = 0.04). Similarly, the group by condition by total PSS interaction on right amygdala activity remained significant when covarying tobacco use (*p* = 0.05), positive toxicology screen (*p* = 0.03), and total HDRS scores (*p* = 0.03). There was no significant relation between time since first manic episode and right amygdala response to stress in bipolar disorder (β = −0.005, *p* = 0.9).

### Amygdala Reactivity During SMT and Relations With Alcohol/Cannabis Use

**Figure 2:**
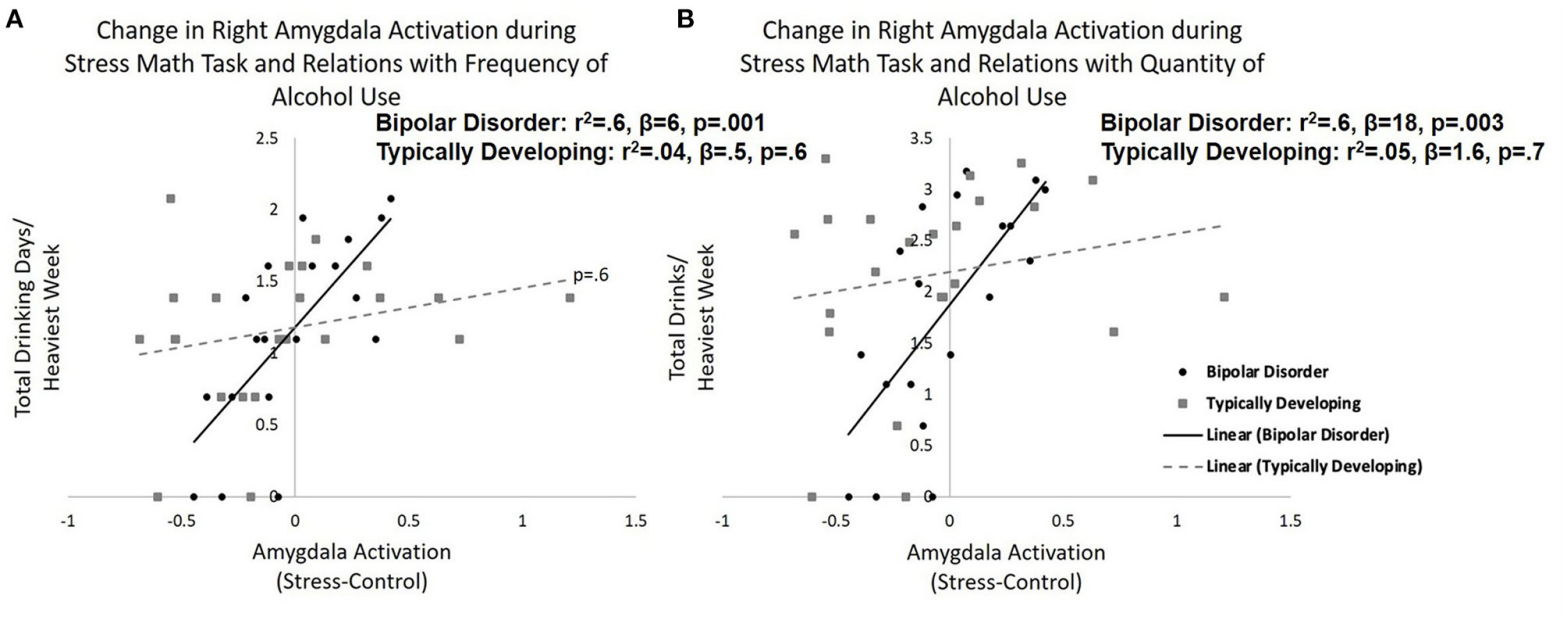
Amygdala reactivity to acute psychosocial stress and relations with recent alcohol use. Relations between right amygdala reactivity to acute psychosocial stress [stress condition minus control condition during stress math task (SMT)] in typically developing young adults and young adults with bipolar disorder and recent **(A)** frequency (total drinking days during heaviest drinking week) and **(B)** quantity (total drinks during heaviest drinking week) of alcohol use. Specifically, greater right amygdala reactivity to acute stress correlated with greater frequency (*p* = 0.001) and quantity (*p* = 0.003) of alcohol use in young adults with bipolar disorder. There was no significant relationship between amygdala reactivity and alcohol use in typically developing adolescents/young adults.

When investigating group and calculated amygdala reactivity to stress interactions that may be predictive of recent alcohol use the multiple regression model for total days drinking per heaviest week was significant (*r*^2^ = 0.34, *F* = 3.7, and *p* = 0.008). Specifically, there were significant group by change in right amygdala activation interaction on total drinking days per heaviest week (β = 2.7, *p* = 0.002). When stratifying by group, the model was significant in bipolar disorder (*r*^2^ = 0.57, *F* = 6.6, and *p* = 0.005) with increased right amygdala activation in response to acute stress associated with greater drinking days (β = 5.7, *p* = 0.001; Figure 2A). Amygdala activation was not associated with total drinking days in typically developing youth (β = 0.48, *p* = 0.6). A similar significant group by change in right amygdala activation interaction on total drinks per heaviest drinking week was observed (β = 8.7, *p* = 0.02), but the overall model for total drinks per heaviest drinking week did not reach significance (*r*^2^ = 0.24, *F* = 2.3, and *p* = 0.06). When stratifying by group, the model was significant in bipolar disorder (*r*^2^ = 0.56, *F* = 6.4, and *p* = 0.005) with increased right amygdala activation in response to acute stress associated with greater total drinks (β = 18.4, *p* = 0.003; Figure 2B). Amygdala activation was not related to total drinks in typically developing youth (β = 1.6, *p* = 0.7). Similarly, there was a group by change in right amygdala activation interaction on number of days using cannabis during the heaviest drug-taking week (β = 3.4, *p* = 0.009), but the overall model for days smoking cannabis did not reach significance (*r*^2^ = 0.22, *F* = 2.1, and *p* = 0.09). When stratifying by group, the bipolar group showed a significant positive relationship between right amygdala activation to the stress condition and frequency of cannabis use (β = 5.5, *p* = 0.03). Amygdala response to the stress condition was not related to frequency of cannabis use in the typically developing group (β = −0.6, *p* = 0.6).

## Discussion

Findings support our hypothesis that recent perceived stress is associated with greater amygdala reactivity to acute stress in adolescents/young adults with bipolar disorder early in their illness course compared to typically developing adolescents/young adults. Specifically, adolescents/young adults with bipolar disorder showed a positive relationship between past month perceived stress and amygdala reactivity to the stress condition, compared to the control condition, of the SMT. Also, in line with our predictions, we observed greater amygdala reactivity to the stress condition related to greater frequency and quantity of alcohol use, and greater frequency of cannabis use in bipolar disorder. Typically developing adolescents/young adults did not show an association between recent perceived stress and amygdala reactivity to the acute psychosocial stress fMRI task, nor did they exhibit relations between amygdala reactivity during the SMT and recent alcohol/cannabis use. Findings suggest variation in stress response may relate to alcohol/cannabis use during adolescence/young adulthood in bipolar disorder and may serve as a target for early intervention.

Bipolar disorder is progressive and stress response is thought to sensitize with age in bipolar disorder (21). While there were no between-group differences in amygdala reactivity (27), it is possible greater between-group differences in amygdala response to stress will emerge over time, although we did not observe main effects of age or group by age interactions on amygdala reactivity to the stress condition. While we can only speculate, stress may potentiate amygdala reactivity in adolescents/young adults and over time group differences in stress-induced amygdala reactivity may become more robust. Additionally, interactions between alcohol/substance use and stress may contribute to neuroadaptations that subsequently alter activation by alcohol/substance use and stress—as alcohol/substance use and stress are known to cross-sensitize (39, 40)—and ultimately confer risk for alcohol/substance use disorders. Recent alcohol/cannabis use also did not differ between groups. However, the bipolar disorder group did report higher past month perceived stress compared to the typically developing group. Higher perceived stress may have contributed to positive findings in bipolar disorder and null findings in the typically developing group. Berghorst et al. (17) reported differences in amygdala response when investigating the influence of stress on amygdala activity during reward processing in 13 adults with bipolar disorder, compared to 15 healthy adults, and that recent perceived stress was not associated with variation in amygdala activation. While fMRI tasks differed between the Berghorst study and the current study, other clinical factors, including differences in age (adults being studied vs. adolescents/young adults), may have also contributed to the lack of association between recent perceived stress and amygdala reactivity. For example, recent perceived stress did not differ between groups in the prior study, and was lower, on average, compared to what is reported here in the bipolar disorder group. Additionally, the current study had a larger percentage of females. Sex differences are suggested in stress response and stress-related alcohol use (41, 42) and in pathways that contribute to risk for alcohol/substance use disorders (43–46), including in bipolar disorder (10). Specifically, there is evidence for females drinking/smoking for negative reinforcement (i.e., stress, negative mood, and depressive symptoms) and males drinking/smoking for positive reinforcement (i.e., social, enhancement motives) (42, 46). Future studies, with larger sample sizes, the power to investigate sex interactions, and groups matched on recent perceived stress (including higher levels of perceived stress), are needed to extend findings and contribute to our understanding of similar/distinct stress response in bipolar disorder and typically developing peers. Additionally, such studies could further probe biological factors, including genetic vulnerability (47), that may mediate the relationship between stress, amygdala reactivity to acute stress, and alcohol/cannabis use.

We did not originally hypothesize right lateralization of findings. While right lateralization of dysfunction is suggested in bipolar disorder, including evidence from lesion studies, results are mixed (48). Studies also suggest left and right lateralization of dysfunction may be dependent on mood state as previously described (48). More research is needed to disentangle lateralization of dysfunction in bipolar disorder, including state-related differences, and if lateralization may be specific to subtypes of bipolar disorder, e.g., those exposed to high levels of stress. Mothersill and Donohoe (49) reported the right amygdala was most frequently observed to be associated with high levels of environmental stress in a meta-analysis of 54 functional MRI studies.

Several limitations should be noted. As previously discussed (27), findings should be considered preliminary. We employed a ROI approach to test our *a priori* clinical hypothesis and balance study sensitivity while also reducing the number of comparisons performed (50). As recommended for ROI approaches (51), bilateral amygdala ROI selection predated data analysis, was defined anatomically, and was chosen as our a priori ROI based on a large body of evidence—including meta-analyses (49, 52–55)—supporting altered function of the amygdala in bipolar disorder, alcohol use disorders, and stress response. However, we cannot rule our false positives and future research is needed to confirm and extend these preliminary findings. We cannot rule out increased cognitive load during the stress condition of the SMT may have contributed to findings. Higher hair cortisol concentrations in people with bipolar disorder are associated with poorer cognitive performance (56), suggesting that while cognitive performance may not have biased fMRI findings, amygdala reactivity during acute stress may interfere with cognitive performance. More work on how stress impacts cognition in bipolar disorder is needed as deficits in executive functions are reported in bipolar disorder and may relate to risk for alcohol/substance use disorders (57, 58). It is possible groups differed in how stressful they perceived the task. Greater amygdala metabolism has been shown to correlate with cortisol in bipolar disorder; however, we did not measure cortisol. While both groups showed a similar increase in heart rate to the stress condition, future studies should investigate other physiological markers, i.e., stress hormones and heart rate variability (59). We were underpowered to investigate sex differences. Individuals with bipolar disorder also reported greater past month tobacco use and had greater depression symptoms. Rates of current tobacco use were low for both groups and only those with mild depression symptoms over the past week were included in this study. When covarying tobacco use and HDRS scores, results remained significant. Future work is needed to investigate effects of tobacco, including specifically investigating electronic cigarette use and nicotine vaping, on amygdala activity to psychosocial stress. Similarly, many individuals in both groups had positive urine toxicology screens, especially for tetrahydrocannabinol (THC). The majority of individuals with bipolar disorder (74%) were medicated at the time of their scan. We were underpowered to investigate medication or cannabis use interactions on stress-response. Additionally, future larger powered studies should extend investigation beyond the amygdala to include other regions involved in stress response as well as executive functions.

Despite these limitations, this study contributes to evidence suggesting variation in neural responses to stress may contribute to alcohol/cannabis use in adolescents/young adults with bipolar disorder. This study suggests differences in stress response in bipolar disorder, even at a younger age than previously observed, may serve as a modifiable target for early prevention. While results should be considered preliminary, they support future, larger powered studies that can tease apart the role(s) of the amygdala, and regions outside of our a priori ROI, in stress sensitization in bipolar disorder and risk for alcohol use disorders. An additional next step would include longitudinal studies designed to examine the impact of brain development to better understand long-term outcomes, e.g., hyperactive stress system and development of alcohol/substance use disorders, and modifiable targets to mitigate these outcomes.

## Data Availability Statement

The raw data supporting the conclusions of this article will be made available by the authors, without undue reservation.

## Ethics Statement

The studies involving human participants were reviewed and approved by the University of Texas at Austin Institutional Review Board. The patients/participants provided their written informed consent to participate in this study.

## Author Contributions

VL, DK, VT, and EL: study concept, design, data analysis, and interpretation. DK, VT, VL, WW, and EL: data acquisition. VL, DK, and EL: drafting of manuscript and statistical analyses. VL, DK, VT, WW, SS, and EL: critical revisions of manuscript, administrative, technical, and material support. EL: obtained study funding and study supervision. All authors contributed to the article and approved the submitted version.

## Funding

The authors were supported in part by research grants from NIAAA K01AA027573 (EL), R21AA027884 (EL), T32AA007471 (VT), and Jones/Bruce Fellowship from the Waggoner Center on Alcohol and Addiction Research (DK and VT).

## Conflict of Interest

SS and EL received funding for a Janssen-sponsored study through UT. SS serves as DSMB chair for Sunovion, and served recently on a DMC for Otsuka. He is also a contributor to Medscape. The remaining authors declare that the research was conducted in the absence of any commercial or financial relationships that could be construed as a potential conflict of interest.

## Publisher's Note

All claims expressed in this article are solely those of the authors and do not necessarily represent those of their affiliated organizations, or those of the publisher, the editors and the reviewers. Any product that may be evaluated in this article, or claim that may be made by its manufacturer, is not guaranteed or endorsed by the publisher.
